# On the Poison of Arsenic

**Published:** 1817-12

**Authors:** W. J. Crowfoot


*For the London Medical and Physical Journal.*

### *On the Poison of Arsenic;* by Mr. W. J. Crowfoot.

As I observe, in your Journal for August, a paper, by Mr. Kerr, on Poison by Arsenic, in which he adverts to some experiments of mine mentioned in a communication of December, 1815, I beg leave to make a few remarks on the subject. Mr. K. considers the nitrate of silver as no longer admissible as a test for the arsenious acid, from the circumstance circumstance of the phosphate, as well as the arsenite of silver, being precipitated of a yellow colour. Upon the first precipitation of these two salts, I allow, there is a great resemblance; but, if we suffer them to remain undisturbed and exposed to the light for a few hours, the difference will be very observable,—the former becoming gradually of a brown colour, while the latter assumes a blackish hue. To satisfy myself still further on the subject, I made the following experiments:—

Exp. I.—To a solution of arsenic was added a watery solution of sulphuretted hydrogen. The liquid was immediately changed to the well-known yellow colour, and after some hours a precipitation took place.

Exp. II.—To a solution of phosphate of soda was added, as in the preceding experiment, the sulphuretted hydrogen; but no change was perceptible.

*Walworth; Sept. 8th.*

